# Tapia’s Syndrome (Concurrent Unilateral Recurrent Laryngeal and Hypoglossal Nerve Palsy) Following Left Retrosigmoid Craniotomy for Schwannoma Resection

**DOI:** 10.7759/cureus.17909

**Published:** 2021-09-12

**Authors:** Clara R Stelman, William Buxton, Jeffrey D Sharon

**Affiliations:** 1 Department of Anesthesiology and Perioperative Medicine, University of California San Francisco, San Francisco, USA; 2 Department of Otolaryngology Head and Neck Surgery, University of California San Francisco, San Francisco, USA

**Keywords:** tapia's syndrome, orotracheal intubation, vocal cord paralysis, diagnostic fibroscopy, airway management, hoarseness, hypoglossal nerve palsy, recurrent laryngeal nerve palsy, tongue deviation

## Abstract

Tapia’s syndrome, a unilateral, extracranial combined lesion to the hypoglossal nerve (cranial nerve [CN] XII) and the recurrent laryngeal branch of the vagal nerve (CN X), has been observed to occur after general anesthesia for a variety of surgical procedures. Surgical intraoperative neck positioning and airway management are hypothesized as causative factors. The condition presents with ipsilateral motor paralysis of the tongue and vocal cords. Postoperatively, patients often present with dysphonia, dysphagia, and difficulty swallowing.

We discuss a unique case of Tapia’s syndrome occurring after retrosigmoid craniotomy for left vestibular schwannoma resection in a 42-year-old male. General anesthesia was uneventful with an atraumatic, grade 2a intubation and a normal endotracheal tube cuff pressure of 30 cm of water. The patient was positioned laterally, even though the exact head position was not documented. Institutional practice in these cases is for the head to be maintained neutral or with a slight turn. An uneventful subtotal resection of the tumor was performed after retrosigmoid exposure.

Postoperatively, the patient complained of left-sided mouth tingling, a hoarse voice, and tongue weakness which impacted his ability to chew and swallow. He had mild left-sided facial weakness and decreased sensation in the V1 and V2 distribution of the trigeminal nerve. Postoperative brain MRI showed postsurgical changes without evidence of neurological or vascular involvement.

Fiberoptic endoscopy performed in the otolaryngology clinic showed immobility of the right vocal cord. Consequently, Tapia’s syndrome was diagnosed. He later underwent a right vocal fold injection with Prolaryn gel (Merz North America, Inc, Greensboro, NC, USA) via flexible laryngoscopy with a slight improvement in his dysphonia. At his last visit, he declined further interventions based on acceptable voice quality.

Tapia’s syndrome can occur due to the close anatomical proximity of the hypoglossal and recurrent laryngeal nerves as they pass lateral to the oropharynx and hypopharynx. This predisposes the nerves to anesthetic and surgical insults such as over-stretching of the nerves during head manipulation and trauma to the nerve fibers following laryngoscopy.

Our case report highlights this potential rare complication to anesthetic and surgical teams. Awareness of this concurrent paralysis can assist practitioners to rapidly diagnose and treat patients who present in this way postoperatively. It can also enable avoidance of causative factors and remind practitioners of the importance of meticulous perioperative documentation.

## Introduction

First described in 1904 by Antonio Garcia Tapia, Tapia’s syndrome is a rare but known complication of perisurgical neck positioning and/or airway management [1-3]. It is caused by unilateral extracranial, combined lesions to the hypoglossal nerve (cranial nerve [CN] XII) and the recurrent laryngeal branch of the vagal nerve (CN X) [4]. According to an extensive literature search, it can occur in a variety of surgeries [2,5-19], and general anesthesia is a common factor among all previously described cases. The condition presents with ipsilateral motor paralysis of the tongue and vocal cords. Postoperatively, patients often complain of dysphonia, dysphagia, and difficulty with swallowing. Tapia’s syndrome occurring after retrosigmoid craniotomy has not been previously reported in the literature, making this a unique case.

## Case presentation

**Figure 1:**
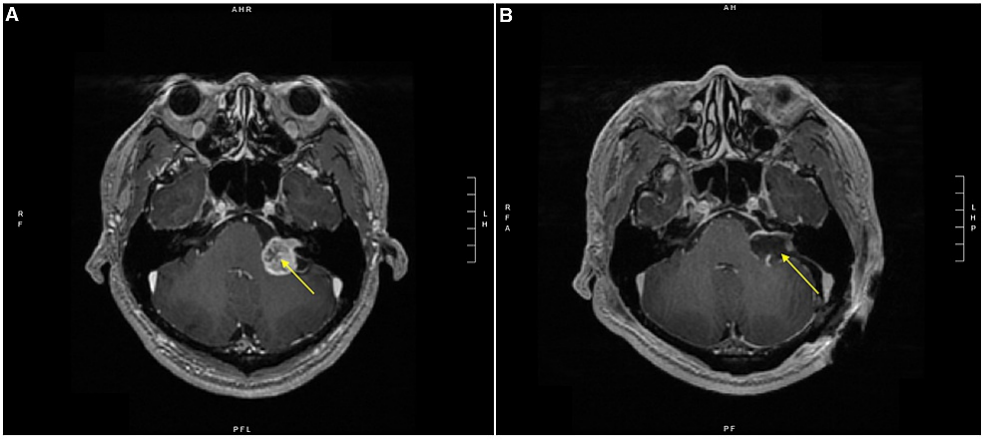
T1-weighted brain MRI. (A) Preoperative brain MRI with a yellow arrow pointing to the vestibular schwannoma in the left cerebellopontine angle. (B) Postoperative brain MRI with a yellow arrow pointing to postsurgical changes following debulking of the left vestibular schwannoma and a residual ring of enhancing tumor. MRI: magnetic resonance imaging

A 42-year-old male originally presented to our hospital with persistent left-sided hearing loss. Brain MRI showed an enhancing cerebellopontine angle mass consistent with left vestibular schwannoma (Figure 1, Panel A). The patient was taken to the operating room for left retrosigmoid craniotomy for tumor resection under general anesthesia. Anesthesia was induced with standard induction doses of propofol, 2% lidocaine, and fentanyl while rocuronium was used for paralysis. Endotracheal intubation was achieved following a single attempt with a Macintosh 3 laryngoscope, and a 7.5 mm endotracheal tube was inserted into the trachea. A Cormack-Lehane grade 2a view was recorded. The endotracheal tube cuff was inflated with 8 mL of air to achieve cuff pressure of 30 cm of water, as measured by a manometer. The endotracheal tube was secured at 24 cm at the teeth and taped in place. Overall, airway management was unremarkable. Anesthesia was maintained with 0.3 minimum alveolar concentration of a volatile anesthetic (MAC) of sevoflurane and a propofol infusion which commenced at 150 µg/kg/min and was reduced in a stepwise manner throughout the case. Nitrous oxide was not used. A Mayfield pin headrest was applied to the patient’s head after which he was turned laterally (right side down) on the operating room table. It is not documented whether the head was secured in a neutral position; however, either a neutral position or a slight tilt on the head is the usual practice for such procedures in our institution.

Neuromonitoring was undertaken by a neurophysiologist. Monitoring of CN X and CN XII was not requested by the surgeon and thus was not conducted. CN VII and CN VIII (auditory brainstem response) were requested and performed, along with long tract monitoring with somatosensory evoked potential. The remnant vestibulocochlear nerve (CN VIII) was sacrificed during the case. The facial nerve (CN VII) had a low threshold of stimulation at 0.1 V at the end of the operation, indicative of a healthy nerve.

An uneventful subtotal resection of the tumor was performed after retrosigmoid exposure. After initial tumor exposure and debulking, the facial and vestibulocochlear nerves were exposed at the root entry zone. The internal auditory canal was exposed, allowing for lateral identification of the facial, cochlear, and vestibular nerves. The latter two were intentionally sacrificed, which allowed for resection of nearly the entire tumor, except for a small adherent ring on the facial nerve. A multilayered closure was performed, with dural closure, titanium cranioplasty, and approximation of the muscle, dermis, and skin. Total anesthesia time was 576 minutes and total operation time was 470 minutes. The patient was spontaneously breathing and conscious prior to extubation in the operating room and was immediately transferred to the neurosurgical intensive care unit (ICU) for recovery.

**Figure 2:**
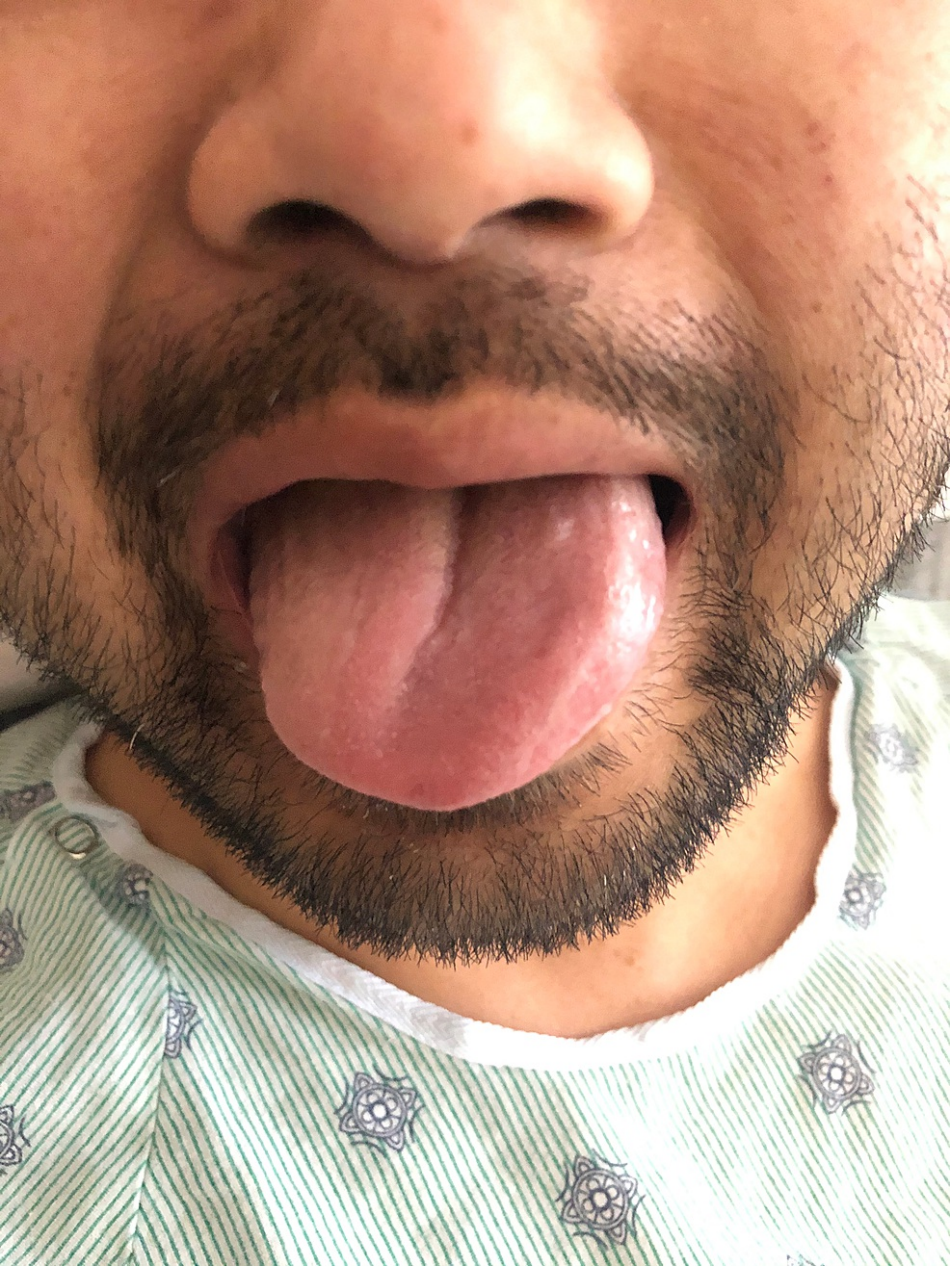
Rightward tongue deviation on attempted midline protrusion, indicative of right hypoglossal nerve injury.

Upon arrival in the ICU, the patient endorsed left-sided mouth tingling. He was noted to have a hoarse voice and tongue weakness which impacted his ability to chew and swallow. His presentation raised an immediate concern for an intraoperative stroke. Postoperative brain MRI showed postsurgical changes without evidence of neurological or vascular involvement (Figure 1, Panel B). Neurological examination was consistent with tongue deviation to the right upon attempted midline protrusion (Figure 2), mild left-sided facial weakness, and decreased sensation in the V1 and V2 distribution of the trigeminal nerve.

Right side hypoglossal nerve and recurrent laryngeal nerve palsy were diagnosed, consistent with Tapia’s syndrome. The patient was discharged on postoperative day two with acetaminophen for pain control and a dexamethasone taper. During the following weeks, the hypoglossal nerve palsy resolved; however, he continued to experience dysphonia. No symptoms of aspiration were reported. Fiberoptic endoscopy was performed in the otolaryngology clinic and showed immobility of the right vocal cord (Video 1). He later underwent a right vocal fold injection with Prolaryn gel (Merz North America, Inc, Greensboro, NC, USA) via flexible laryngoscopy with a slight improvement in his dysphonia. At the time of writing, the patient continues to follow up with the laryngology clinic, and has not yet had normal return of recurrent laryngeal nerve function. At the last visit, he declined further interventions based on acceptable voice quality.

**Video 1:** Video of right vocal cord palsy.

## Discussion

Tapia’s syndrome, a unilateral, combined extracranial palsy of the hypoglossal and recurrent laryngeal nerves, is a rare but well-described complication of endotracheal intubation [5,6,18,19]. Unilateral paralysis of the vocal cords and tongue causes affected patients to experience voice hoarseness, difficulty in moving their tongue, and dysphagia. An extensive review of the literature shows that numerous cases of Tapia’s syndrome have been described, generally affecting patients who required airway manipulation for general anesthesia [1,2,4,5,7-17,19]. Rhinoplasty and septorhinoplasty were two of the most frequently described procedures associated with Tapia’s syndrome [5,11,13]. However, several cases following orthopedic, cardiac, and maxillofacial surgeries involving extreme neck positioning have also been reported [14,15]. No cases of Tapia’s syndrome have been described in patients undergoing retrosigmoid craniotomy. Patients are generally treated in a supportive manner, and, occasionally, severe nerve damage can warrant a course of systemic corticosteroids [12]. Most patients are reported to make a full recovery within a few months.

The hypoglossal nerve (CN XII) controls the intrinsic and extrinsic muscles of the tongue. It starts in the hypoglossal nucleus of the medulla and exits the cranial cavity through the hypoglossal canal. It passes down the neck between the internal carotid artery and the internal jugular vein. On the lateral aspect of the tongue, it lies close to the hyoglossus muscle and inferior to the lingual nerve. The recurrent laryngeal nerve, a branch of the vagus nerve (CN X), ascends within a groove between the trachea and esophagus to reach the larynx, supplying all of the intrinsic muscles of the larynx except the cricothyroid muscle. The hypoglossal and recurrent laryngeal nerves are located adjacent to the oropharynx junction and the hypopharynx, thus concurrent injury and paralysis are most likely in this area [18].

Damage to these nerves can result in either neuropraxia (temporary loss of function) or axonotmesis (usually permanent, irreversible damage). An excessive stretch of the hypoglossal nerve from neck manipulation can occur on the anterior surface of the first cervical transverse process as it intersects the vagus nerve, causing Tapia’s syndrome. Suspected causative factors that have been highlighted in other case reports for Tapia’s syndrome include placement of a throat pack or neck manipulation intraoperatively, nerve impingement by the endotracheal tube, overinflation of the endotracheal tube, extubation with the cuff inflated, and trauma to the nerve fibers following laryngoscopy [6,12,16,17,19].

In our patient, we have identified several factors which we hypothesize may have caused this unilateral hypoglossal and recurrent laryngeal nerve palsies following his retrosigmoid craniotomy.

Male patients are generally more likely to develop this syndrome due to, on average, having larger hyoid bone dimensions, which may cause the nerves to become impinged in certain positions with airway instrumentation. It is also possible that our patient may have had anatomical abnormalities which predisposed him to injury from positioning or intubation. The amount of time spent under general anesthesia may have also played a role in the development of neuropraxia in this patient. Other potential causes include possible tube migration after head manipulation for surgical site exposure which may have caused unfavorable soft tissue and nerve compression. Pressure from the cuff may have been a causative factor, though it was documented to have only been inflated to 30 cm of water which is generally considered a safe level. Finally, even though laryngoscopy was largely unremarkable and tube placement was achieved on the first attempt, the direct pressure exerted by the blade at the base of the tongue may have caused nerve shearing and soft tissue compression, leading to the observed neuropraxia.

This case demonstrated the importance of good perioperative documentation pertaining to the patient’s position, the angle of the neck after being placed in the head brace, endotracheal tube size, depth, securing technique, the intubation itself, and cuff pressure. The electronic medical record prompts anesthesiologists to record these details. In our case, this ensured that meticulous documentation was kept enabling the team to review in detail all aspects of airway manipulation and to assess whether a specific causative factor could be identified.

## Conclusions

Tapia’s syndrome can cause significant challenges to patients affected by it. As the most common mechanism of injury is neuropraxia, most patients make a full recovery within a few months. However, like in our patient’s case, some continue to experience residual symptoms due to more severe nerve injuries. This report aims to demonstrate the need for anesthesia providers and surgeons alike to familiarize themselves with Tapia’s syndrome, its causes, and the fact that it may occur despite seemingly uneventful airway management and neck positioning. By being aware of this rare complication, we hope that practitioners may be able to reduce its occurrence. Anesthesiologists may consider discussing the risk of nerve palsies with their patients and, from a legal standpoint, review their own practice regarding documentation of intraoperative airway management and positioning.
